# Spontaneous Ureteric Rupture and Its Implications in the Emergency Department: A Case Report

**DOI:** 10.5811/cpcem.2021.2.50652

**Published:** 2021-03-12

**Authors:** Tallie Wei Lin Chua, Evelyn Wong

**Affiliations:** Singapore General Hospital, Department of Emergency Medicine, Outram Road, Singapore

**Keywords:** Spontaneous ureteric rupture, urolithiasis

## Abstract

**Introduction:**

Spontaneous ureteric rupture is uncommon and has a wide range of presentations. Accurate diagnosis and timely treatment is necessary to avoid potential serious complications.

**Case Report:**

We present the case of a 55-year-old female who presented with severe right lower abdominal pain with rebound tenderness, vomiting, and a single episode of hematuria. A computed tomography with intravenous contrast of the abdomen and pelvis showed a 0.3-centimeter right upper ureteric calculus, with hydronephrosis and ureteric rupture. In view of the scan findings, a diagnosis of spontaneous ureteric rupture secondary to urolithiasis was made. The patient underwent a percutaneous nephrostomy and ureteric stenting.

**Conclusion:**

Spontaneous rupture of the ureter is an uncommon diagnosis for which clinical and laboratory signs may not always be reliably present. A high index of suspicion is required for diagnosis, which is usually confirmed on advanced imaging. It may occur in serious complications of urinoma and abscess formation. As such, accurate diagnosis and timely treatment is crucial.

## INTRODUCTION

Spontaneous ureteric rupture, or extravasation of the urine from the ureter in the absence of trauma or iatrogenic ureteric manipulation, is a rare condition. The literature, especially in emergency medicine, is fairly sparse. It may mimic many other causes of acute abdomen and itself requires prompt treatment. Thus, it is a diagnosis for which emergency physicians (EP) should have a high index of suspicion. We report a case of spontaneous ureteric rupture in the presence of a small, obstructing ureteric calculus. Informed consent was obtained from the patient for publication of this case report.

## CASE REPORT

A 55-year-old female presented to the emergency department (ED) with acute onset right lower abdominal pain a few hours prior. The pain was non-radiating, constant, and gradually increasing in intensity. She reported one episode of gross hematuria at home with the onset of the pain but the hematuria resolved subsequently. The patient had vomiting with the pain but did not have any other gastrointestinal symptoms. She did not have any fever or chills.

The patient had no past medical problems but a previous surgical history of a total hysterectomy and bilateral salpingo-oopherectomy performed four years prior and a laparoscopic procedure converted to open deroofing of a hepatic cyst three years prior. During a computed tomography (CT) of the abdomen and pelvis ordered by her hepatobiliary surgeon three years prior, a small, right kidney mid-pole stone was noted. However, as it was not causing any symptoms and was relatively small in size, it was conservatively managed.

On physical examination in the ED, she was afebrile and hemodynamically stable. Examination of the abdomen revealed tenderness over the right flank and iliac fossa with rebound tenderness but no guarding.

Initial blood investigations showed a total white cell count of 11×10^3^ per microliter (*μL)* (reference range 4.5 – 11.0×10^3^/*μL*) with mild neutrophilia. Serum electrolyte and creatinine levels were within normal range. Urinalysis showed microscopic hematuria with no casts. Point-of-care ultrasound did not reveal any intraperitoneal free fluid or the presence of an abdominal aortic aneurysm, and no findings suggestive of cholecystitis.

**Image 1:**
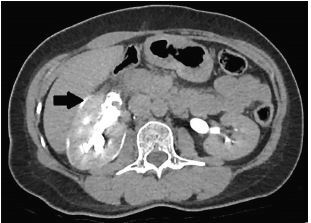
Axial view of computed tomography with intravenous contrast administered showing perinephric leakage (arrow) of contrast showing evidence of ureteric rupture.

**Image 2:**
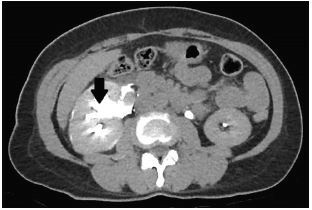
Axial view of computed tomography with intravenous contrast administered showing hydronephrosis (arrow) of the right renal pelvis.

**Image 3:**
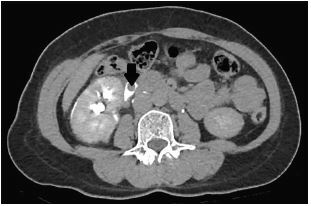
Axial view of computed tomography with intravenous contrast administered, showing filling defect (arrow) in the right ureter suggestive of ureteric stone.

The differential diagnosis in this patient was broad and included appendicitis, renal colic, diverticulitis, etc. However, the presence of rebound tenderness and persistent pain prompted the decision for the patient to undergo a CT of the abdomen and pelvis in the ED. Meanwhile, intravenous ceftriaxone was administered in view of the presence of peritonitis. The contrast-enhanced CT showed no evidence of acute appendicitis but found a 0.3-centimeter right upper ureteric calculus, with upstream hydronephrosis and ureteric rupture (Image 1–3).

**CPC-EM Capsule**

What do we already know about this clinical entity?

*Spontaneous ureteric rupture is a rare urological emergency that has only been described in case reports and series, mainly in surgical and urological literature*.

What makes this presentation of disease reportable?

*Neither symptoms nor simple radiological or lab tests are reliable for diagnosis. Only computed tomography (CT) done in view of peritonitis revealed the diagnosis*.

What is the major learning point?

*Ureteric rupture requires prompt treatment. Providers need to be aware of this condition and when a CT should be done to minimize diagnostic delay*.

How might this improve emergency medicine practice?

*By increasing awareness about this rare disease, emergency physicians may have a higher index of suspicion and be better able to diagnose and manage this emergency*.

Given the imaging findings, the patient was admitted to the urology service. She underwent urgent right percutaneous nephrostomy on the day of admission. Three days later, anterograde ureteric stenting was performed with a confirmatory nephrostogram the following day. She was subsequently discharged with a follow-up CT of the kidneys, ureters, and bladder in three weeks and urology clinic follow-up after the scan.

## DISCUSSION

Spontaneous ureteric rupture is a rare urological emergency, which to our knowledge, has only been described in case reports and case series. The most common cause is lithiasis; other possible etiologies include metastatic invasion of the ureter, urinary retention from neurogenic bladder, connective tissue diseases, retroperitoneal fibrosis,^1^ pregnancy,^2^ ureteral strictures from a variety of causes such as previous instrumentation or radiation, autoimmune, or neoplastic causes.^3^ The majority of the cases described are in the urological and surgical literature. The focus of these articles is often describing various treatment techniques employed, as there are no definitive guidelines for treatment given its rarity.^4^

The clinical presentation of this condition is more relevant to the ED environment, but the existing literature provides little information on this topic. Sudden onset of abdominal or flank pain seems to be common.^5^, ^6^ From case reports, urinary symptoms were not always present, costovertebral angle tenderness was present in some cases but not in others,^7^, ^8^ and signs of peritonitis or fever were also not reliably present in all cases.^9^ In many of the cited cases, laboratory findings such as leukocytosis or elevated inflammatory markers ranged from normal to elevated, which makes these unreliable for diagnosis. Urinalysis also showed mixed results, and hematuria or pyuria was not reliably observed in all cases.

Point-of-care ultrasound detection of urolithiasis and hydronephrosis has limitations,^10^ especially in smaller stones.^11^ Contrast-enhanced CT with normal portal venous and excretory phases confirms the diagnosis and potentially gives information on the level of injury, which would in turn assist in planning treatment.^12^, ^13^ Knowing when to obtain a CT is critical to diagnosing ureteric rupture. Presence of peritonitis, persistent pain, or even recurrence and worsening pain^13^ were among the most commonly cited reasons a CT was ordered in the cited cases. Serial abdominal examinations and regular re-examinations of patients in the ED are hence vital in detecting the aforementioned signs, which would subsequently prompt further investigation and imaging. As urolithiasis is the most common cause of ureteric rupture, it is important that patients observed in the ED for ureteric colic are regularly reviewed. Sudden worsening of pain, unabating pain, or peritonitis in such patients should prompt the EP to re-evaluate the diagnosis of ureteric colic only.

Complications that may arise from this condition, which EPs should be cognizant of, include formation of urinomas, or perinephric or retroperitoneal abscesses. Urosepsis and its attendant risks is also possible following ureteric or perinephric rupture. Thus, prompt treatment and admission of this diagnosis, once made, is necessary.

## CONCLUSION

Spontaneous ureteric rupture is an uncommon urological emergency. Not only are signs and symptoms varied and at times non-specific, laboratory and plain radiographic studies also provide little definitive evidence for diagnosis. Diagnosis is usually only made if emergency physicians order advanced imaging. To avoid potential complications, EPs need to be aware and have a high index of suspicion of this condition to make an accurate and timely diagnosis of ureteric rupture.
